# Annotations

**Published:** 1889-02-23

**Authors:** 


### Annotations.

"Gone in for Pickl s."

"I suppose young —— has joined his father in practice," said a medical husband to his wife a few days ago. "No," answered the lady; "he has given up physic and gone in for pickles." The young doctor in question was about six and twenty years of age, and had had ample opportunities for finding out by experience how he would like medical practice. After qualifying at twenty-two, and practising with his father for a few years in an old-established practice in a good neighbourhood, he had deliberately "cut" physic and selected "pickles." The circumstances are quite of recent date, and the fact is one well worthy of consideration by young men and their "parents and guardians." When a man thinks of emigrating he usually makes some effort to inform himself of the character and possibilities of several new countries; and if one of them presents more than average impediments to reasonable prosperity it is carefully eschewed, and a more favourable one selected. The same care should be exercised in the choice of an occupation. The medical profession, for some reason, has been regarded with unusual favour by young men for the last dozen or score of years. The result is best shown in the actual experience of established practitioners. The father of the young doctor who has just "gone in for pickles" is a man of sixty, but looks seventy-five. He has been in his present practice for nearly forty years. His character is excellent, his abilities are good, he has had money with one of his two wives; and yet such has been his fortune that in spite of the steadiest industry and the most worthy conduct, he is probably poorer at sixty than he was at thirty, and has several children dependent upon him. His prospect is to work on until he can work no longer, and then to be maintained by his children if any of them are in a position to maintain him. If not, then what? Who knows? The outlook is more than unhappy. Another practitioner of sixty, in the same wealthy district, who has been more successful in practice than any of his neighbours, who keeps a pair of horses at the top of their speed from morning till night, who has always been, both personally and in his family affairs, one of the most economical of men, told the present writer, not more than a year ago, that his only prospect was that of 'keeping his nose to the grindstone to the very end of his life." In that same neighbourhood there are shopkeepers, younger by years than these doctors, who doubtless began life at a much lower level, who now live in houses of two or three times the rent of those occupied by the medical practitioners, who keep troops of servants, and to all intents and purposes "roll in wealth." They do not require now to work half as hard as the doctor does. They know nothing of going worn out to bed at midnight, and being called out again at two or three o'clock in the morning to face the biting wind and the snow-covered ground. There is something incongruous and even pathetic in a man with white hair and enfeebled health being compelled to wake out of his needed sleep, and after facing the elements, to sit for weary hours by the sick bed. All this is what is taking place among prosperous general practitioners in good neighbourhoods. What then must be the state of matters among the unprosperous in poor neighbourhoods? Many a medical man's family life is a painful tragedy from beginning to end. He knows well that his sick wife and delicate children are pining and dying for want of fresh air alone. He cannot give it them. He feels his own power of work, and sleep, and endurance, to be slowly but surely diminishing, for no other reason but the want of a little temporary rest and change. He cannot afford either the one or the other. All these things are brought about by the indiscriminate rush of young men into the medical profession. A calling that will not insure to men of extraordinary excellence and industry a modest competence for old age, that insists upon keeping them at work until, like exhausted cab horses, they die in harness—such a profession cannot be in a healthy condition. But this is what medicine has come to, and matters are getting steadily worse. Old medical men ought to be able to raise their fees, and so to secure a sufficient income without excessive labour; but so long as they remain general practitioners they cannot do so. The young man who preferred "pickles to physic" showed his good sense. It is all very well to have a gentleman's occupation; but when it means work that wears out the life, accompanied by partial starvation, it is time to think whether it be not possible to be a gentleman at some calling which may be considered less "genteel" indeed, but which does not include brain exhaustion and starvation among its peculiar merits. We commend "pickles" to the sober consideration of intending medical students, and their parents and guardians.

"The Pace that Kills."

Very few, even of stupid men, would affirm that "full gallop" is the right pace for everyday work. It is all very well for special occasions, but it is the very opposite of well for general use. Most people who take the trouble to read regularly, flatter themselves that they are more or less of philosophers. Those who write as well as read, and particularly those whose daily business is writing, do not for a moment doubt that, whoever may belong to the mere unwashed mob of nobodies, they at least are eddying round the centre where modern light is at at its brightest, and know exactly what is going on. It would be an interesting experiment, if a standard of thought power could by any means be established, to test the thousands of public writers by it, and to demonstrate how many of them were capable of original thought, and of persistent independent action. The proportion would be painfully small; and there's the pity of it. Whether writers think or not, they influence public opinion. If they allow themselves to be "run away with" by the hurrying pace of the times, as they almost universally do, they merely play the part of jockeys who do not know when the "post" is reached, and keep on whipping their horses until they drop. Everybody is conscious of the rush and hurry and strain of modern life in large towns. All those who have the power of preaching, either in newspaper or pulpit, will declaim against it and sigh for better things. Is it worth while either declaiming or sighing, if the declaimer does not try to moderate his own pace? In large towns most professional men are fifty years old in constitution before they are forty in actual age. The doctors, who ought theoretically to know all about it, are quite as foolish as the rest: often, indeed, more foolish. If it be true that few preachers practise their own doctrines, it is quite as certain that few physicians take their own advice. What then has modern scientific progress done for us if it has not taught us sense and the art of reasonable self-management? Can it possibly be true that our grandfathers, nourished mainly on the catechisms and doctrines of old-fashioned religion, had more judgment, more sense of proportion, more practical wisdom, more health and more pleasure in life than we their descendants, who pride ourselves not only on being in the rays of the scientific sun, but some of us on forming even a part of that sun himself? It is a homely test that of practical results, but it is an excellently good one. There is hardly any contravening of its conclusions. There is neither sense nor worth in laziness, but it is doubtful if there is more in runaway madness. A very clever man may be a very great fool. A man gets into the full swing of a thing and he cannot get out of it. Then whatever else he is, he does not possess the faculty of greatness. He may earn bread and butter and a fine house, and troops of—not friends, but dinner-eaters. But if he continues to be driven by the torrent, and never re-asserts his freedom and his independent judgment, the final verdict upon his life will be "failure." The pace not only kills health and life, it kills character and reputation. Right and reasonable life means steady work, sufficient recreation, time for independent thought, and the constant exercise of independent judgment and will. Less than this is not the life of a man, but of a slave and a fool.

